# Decadal Journey of CNT-Based Analytical Biosensing Platforms in the Detection of Human Viruses

**DOI:** 10.3390/nano12234132

**Published:** 2022-11-23

**Authors:** Joydip Sengupta, Chaudhery Mustansar Hussain

**Affiliations:** 1 Department of Electronic Science, Jogesh Chandra Chaudhuri College, Kolkata 700033, India; 2 Department of Chemistry and Environmental Science, New Jersey Institute of Technology, Newark, NJ 07102, USA

**Keywords:** carbon nanotube (CNT), biosensor, virus, SARS-CoV-2, toxicity, biocompatibility

## Abstract

It has been proven that viral infections pose a serious hazard to humans and also affect social health, including morbidity and mental suffering, as illustrated by the COVID-19 pandemic. The early detection and isolation of virally infected people are, thus, required to control the spread of viruses. Due to the outstanding and unparalleled properties of nanomaterials, numerous biosensors were developed for the early detection of viral diseases via sensitive, minimally invasive, and simple procedures. To that aim, viral detection technologies based on carbon nanotubes (CNTs) are being developed as viable alternatives to existing diagnostic approaches. This article summarizes the advancements in CNT-based biosensors since the last decade in the detection of different human viruses, namely, SARS-CoV-2, dengue, influenza, human immunodeficiency virus (HIV), and hepatitis. Finally, the shortcomings and benefits of CNT-based biosensors for the detection of viruses are outlined and discussed.

## 1. Introduction

### 1.1. Carbon Nanotubes and Their Applicability in Biosensing

**Figure 1:**
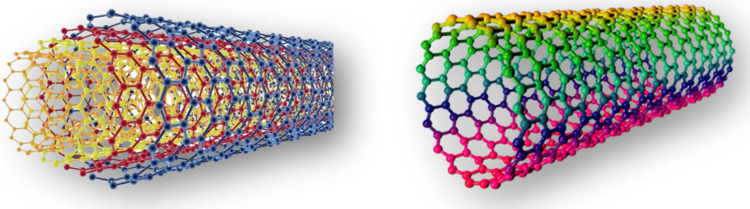
The nanostructure of multi-wall carbon nanotube (MWCNT) (**left**) and single-wall carbon nanotube (SWCNT) (**right**) (adapted with permission from Ref. [3]).

**Scheme 1:**
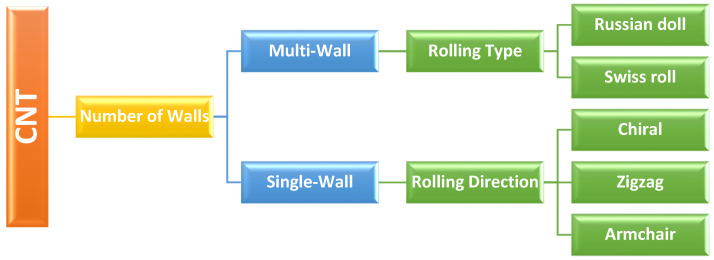
Different categories of carbon nanotube (CNT).

The carbon nanotube (CNT) is the 1D allotrope of carbon; the experimental evidence was first reported by L.V. Radushkevich and V.M. Lukyanovich [1] from the Institute of Physical Chemistry and Electrochemistry of the Russian Academy of Sciences, in 1952. However, after Ijima’s paper [2] in 1991, research interest in CNT escalated rapidly. Structurally, CNTs can be divided into two major categories (Figure 1) based on the number of graphitic layers, namely, single-wall CNT (SWCNT) and multi-wall CNT (MWCNT). Depending on the direction of the roll-up, SWCNTs can have different structures, namely, the zigzag, armchair, or chiral formations. While depending on the nature of the wrapping i.e., whether a graphitic sheet is rolled around itself multiple times (Swiss roll) or if the graphitic sheets are arranged as concentric cylinders (Russian doll), MWCNT can also be categorized, as in Scheme 1.

CNT can be synthesized through various means, such as arc discharge, laser ablation, chemical vapor deposition (CVD), etc. However, because of the scalability, CVD-based approaches appear to be the most appropriate for large-scale CNT synthesis [4]. A wide range of prospective applications in important industrial fields, including nanoelectronics and biotechnology are promised by the distinctive mix of electrical, thermal, mechanical, and chemical characteristics that CNTs display. Additionally, among the many nanomaterials, CNTs are particularly intriguing because they provide an exceedingly tiny inner hollow core, virtually a one-dimensional space, for material storage. Thus, a novel structure can also be formed by filling the core of a CNT with the components necessary for specific applications.

Previous research has discovered a linkage between biomolecules in living beings and illnesses. The monitoring of aberrant physical parameters and the early diagnosis of illnesses help to minimize mortality and ensure organisms’ physical health. Conventional laboratory approaches for assessing pathogenic variables are typically time-consuming, expensive, and complicated. Biosensors can facilitate the reliable and rapid analysis of metabolites in the body to help the current therapeutic procedure. CNTs have unique physicochemical and photoelectric qualities that can improve the performance of biosensors, such as a greater surface area for better catalyst adhesion; CNT-modified electrodes offer quicker electron transfer, resulting in enhanced sensitivity of detection for biosensors. CNT’s unrivaled electronic features, such as quantum wire-like behavior, ballistic-type electronic conduction, remarkable thermal properties derived from phonon quantization, excellent flexibility, and high breaking stress despite its low density make CNT one of the best transducer materials for the transmission of signals related to the recognition of analytes, metabolites, or disease biomarkers. The curvature of the tube contributes to CNTs’ high reactivity and sensitivity to chemical or environmental interactions. Moreover, as the carbon atom near the end of an open-ended tube has only two bonds, foreign molecules can easily enter the structure, thereby helping in the preferential addition of one or more species for functionalization. More importantly, from the viewpoint of biosensors, CNTs can act effectively as scaffolds for the immobilization of biomolecules at their surface. These fascinating characteristics have led to CNTs being widely used in biosensor applications.

### 1.2. Biosensors

**Figure 2:**
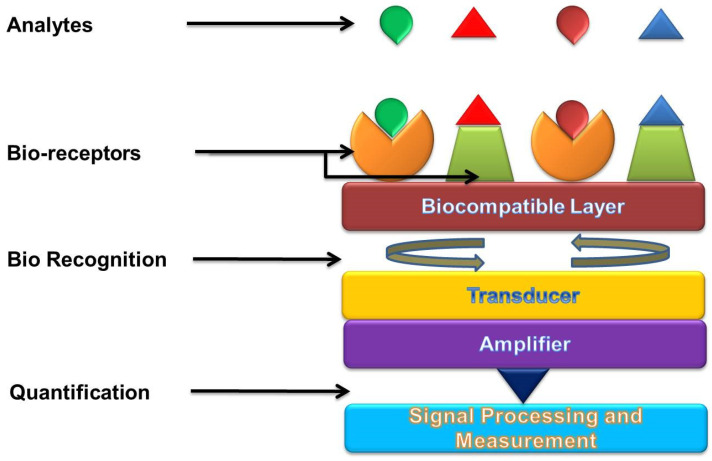
The schematic structure and operating principle of a biosensor.

**Figure 3:**
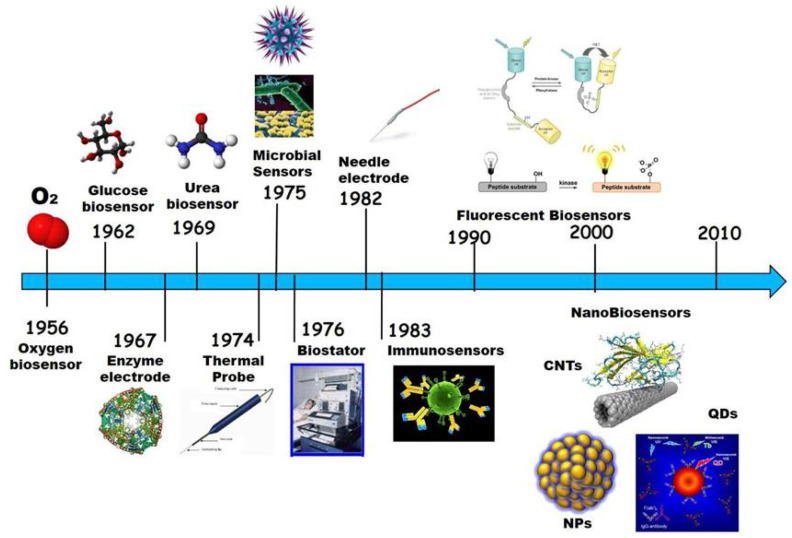
Biosensor development timeline (Reprinted with permission from Ref. [9]).

According to the IUPAC, a biosensor (Figure 2) can be defined as “a device that uses specific biochemical reactions mediated by isolated enzymes, immunosystems, tissues, organelles, or whole cells to detect chemical compounds, usually by electrical, thermal, or optical signals” [4]. In 1962, the first biosensor for monitoring blood glucose was reported by Clark et al. [5]; later, a biosensor was also developed for the detection of the virus [6]. In 1998, Davis et al. [7] were able to immobilize the proteins on CNTs; afterward, in 1999, Balavoine et al. [8] were successful in developing the first biosensor using CNT. The timeline of biosensor development is represented in Figure 3.

**Scheme 2:**
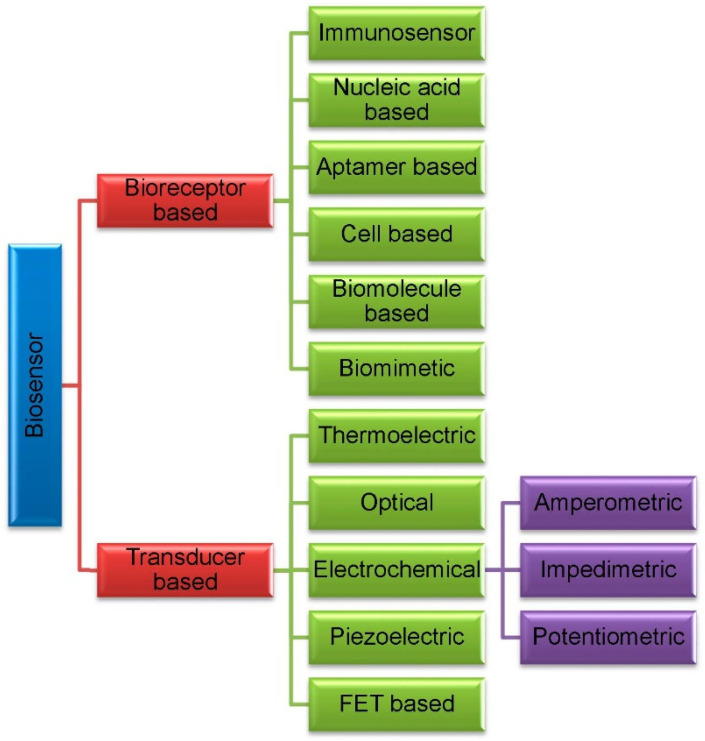
Classification of biosensors, based on analyte and transducer use (adapted with permission from Ref. [10]).

To date, different varieties of biosensors have been fabricated (Scheme 2) based on the analyte and transducer used. However, the type of biosensors that are used for human virus detection falls within the scope of discussion in this review.

## 2. Types of Biosensors Used for Virus Detection

### 2.1. Immunosensors

**Figure 4:**
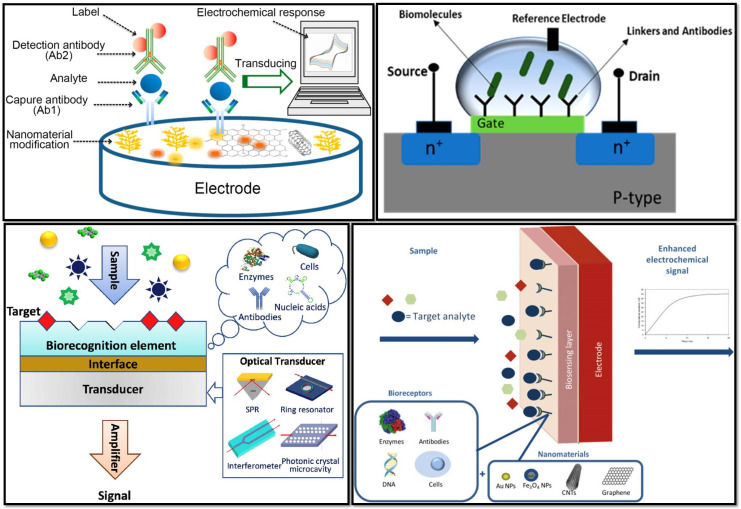
(**Top left**) A schematic representation of an electrochemical immunosensor (Reprinted with permission from Ref. [12]); (**top right**) schematic diagram of a field effect transistor (FET)-based biosensor with a source and drain (Reprinted with permission from Ref. [13]); (**bottom left**) schematic diagram of optical biosensor constitution (Reprinted with permission from Ref. [14]); (**bottom right**) the main constituents of a nanomaterial-based electrochemical biosensor (Reprinted with permission from Ref. [15]).

Because of its capacity to handle information, the immune system is an appealing subject in scientific studies. The major purpose of an immune system, as part of the system’s defensive mechanism, is to accredit and ascertain all cells and molecules in the assembly and classify these biological substances as either toxic or non-toxic. When exposed to foreign substances (i.e., antigens), specialized immune system cells make immunoglobulins (i.e., antibodies) that attach to these antigens precisely. An immunosensor (Figure 4, top left), an affinity-based biosensing device, exploits the concept of immunology and employs an antibody for the specific molecular identification of antigens that are immobilized on a transducer surface, and then develops a stable immunocomplex. The immunocomplex is calculated and quantified by connecting the antibody and antigen interactions to the surface of a transducer. The transducer detects the response and transforms it into an electrical signal, which may then be processed, recorded, and examined. The detection of the target analyte in immunosensors might be direct, by witnessing the production of immunocomplexes, or indirect, by using a label. Immunosensors can be categorized into several categories, based on various methodologies, such as electrochemical, impedimetric, potentiometric, amperometric, voltammetric, conductometric, capacitive, and surface plasmon resonance (SPR)-based methodologies.

### 2.2. Optical Biosensor

An optical biosensor, a compact analytical instrument, combines an optical transducer system with a biorecognition-sensing element (Figure 4, bottom left). An optical biosensor’s primary goal is to provide a signal that is proportional to the concentration of the material being analyzed (the analyte). Optical detection is made possible by using the interplay between the optical field and a biorecognition element. Label-free and label-based optical biosensing are the main two categories of optical biosensors. In a label-free mode, the interaction between the substances is analyzed, and the transducer directly generates the measured signal. In contrast, label-based sensing makes use of a label to assess the biorecognition event and generates an optical signal using a colorimetric, fluorescent, or luminescent approach. However, in some cases, such as antibody-antigen interactions, when a label is coupled with one of the bio-reactants, then this labeling might modify the binding characteristics, introducing systematic inaccuracy into biosensor analysis.

### 2.3. Electrochemical Biosensor

Due to the direct conversion of a biological event to an electrical signal, electrochemical biosensors provide an appealing technique for analyzing the content of a biological sample. The measurement of electrical characteristics in biosensing, for extracting information from biological systems, is generally electrochemical in nature, with a bio-electrochemical component serving as the major transduction aspect (Figure 4, bottom right). While biosensing devices use a variety of recognition components, electrochemical detection approaches mostly involve enzymes. This is mainly owing to their unique binding properties and biocatalytic activity. In bio-electrochemistry, the reaction under examination would typically create a quantifiable current (amperometric), a measurable potential or charge buildup (potentiometric), or a measurable impedance (impedimetric). The electrodes are essential components for the operation of electrochemical biosensors since reactions are generally observed near the electrode’s surface. Depending on the electrode’s parameters, the material, the surface modification, or the electrode’s size have a significant impact on the capability of detection. In general, three electrodes, namely, the reference electrode, counter or auxiliary electrode, and working electrode are needed for electrochemical sensing. To maintain a known and constant voltage, the reference electrode is kept away from the reaction site. The counter electrode creates a link to the electrolytic solution so that a current may be supplied to the working electrode, while the working electrode acts as the transduction element in the biological reaction. These electrodes ought to be chemically stable and conductible to achieve a faithful analysis.

### 2.4. Field-Effect Transistor (FET)-Based Biosensor

FET biosensors, which have the characteristics of being quick, inexpensive, and straightforward, stood out among a wide spectrum of electrical sensing devices as one of the most promising options for biosensing (Figure 4, top right). This cutting-edge technology, which has evolved since 1970 [11] in various forms, is the easiest method for the quick and accurate detection of numerous analytes. Specific probes on the conducting channel of FET-based biosensors can be embedded to provide real-time and label-free analysis. A FET is a type of solid-state device that controls the semiconductor’s electron conductivity between its source and drain terminals by the application of a third gate electrode, via an insulator. To recognize specific analytes, biological receptors are immobilized on the sensing channels, which are linked to the source and drain electrodes. After exposing the biosensor to target analytes and forming specific biological complexes, the transducer system converts biochemical changes into a measurable signal. The addition of charged biomolecules to the surface of the gate dielectric is equivalent to the application of voltage by the use of a gate electrode and results in threshold voltage variations. Therefore, the FET biosensors’ underlying method relies on the conductance of the species that have been adsorbed. The two main types of FETs are n-type and p-type devices, wherein electrons and holes, respectively, serve as the principal charge carriers. An n-type FET sensor will respond by increasing the conductance if the target molecule is positively charged as a result of electron aggregation. Conversely, the conductance will be reduced if the target is a molecule with a negative charge. When it comes to the p-type FET system, the opposite tendency is applicable.

## 3. CNTs-Based Biosensors for the Detection of Human Viruses

### 3.1. SARS-CoV-2

**Figure 5:**
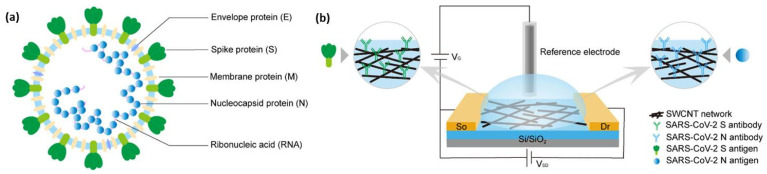
The detection of SARS-CoV-2 Ag, using SWCNT-based FET biosensors. (**a**) Schematic structure of SARS-CoV-2 to demonstrate the targeting proteins. (**b**) Schematic illustration of a liquid-gated SWCNT FET for the detection of SARS-CoV-2 SAg and NAg. Interdigitated gold electrodes (yellow blocks) are configured as the source (So) and drain (Dr) (Reprinted with permission from Ref. [17]).

The SWCNT-based optical sensing approach was employed by Pinals et al. [16] to detect the SARS-CoV-2 spike protein. The angiotensin-converting enzyme 2 (ACE2), which has a strong affinity with the SARS-CoV-2 spike protein, was used to functionalize SWCNTs. A 2-fold increase in fluorescence was observed within 90 min of SARS-CoV-2 spike protein exposure, which exhibited a limit of detection (LOD) of 12.6 nM for the device. Shao et al. [17] functionalized a high-purity semiconducting SWCNT surface with an anti-SARS-CoV-2 spike protein antibody and used it as a channel in an FET-type biosensor, to detect SARS-CoV-2 antigens in clinical nasopharyngeal samples. The fabricated device exhibited an LOD of 0.55 fg/mL (Figure 5). For efficient and precise identification of the SARS-CoV-2 S1 antigens in fortified saliva samples, Zamzami et al. [18] created a rapid, simple-to-use, inexpensive, and quantitative CNT-based antibody-functionalized p-type depletion FET biosensor. Through a non-covalent interaction with the linker 1-pyrenebutanoic acid succinimidyl ester (PBASE), the SARS-CoV-2 S1 antibody was bound to the CNT surface in the FET channel region. The CNT-FET biosensor successfully identified the SARS-CoV-2 S1 antigen in 10 mM AA buffer at a pH of 6.0, at concentrations ranging from 0.1 fg/mL to 5.0 pg/mL, with an LOD of 4.12 fg/mL. On a flexible Kapton substrate, Thanihaichelvan et al. [19] created CNT-FETs and immobilized the reverse sequence of the SARS-CoV-2 RNA-dependent RNA polymerase gene onto the CNT channel, to develop a biosensor for coronavirus detection with an LOD of 10 fM. The primary signal generation depended on RNA hybridization, while the main signal transducer was a liquid-gated CNT-FET. Cardoso et al. [20] used carboxylated CNTs and screen-printed (SP) them onto carbon electrodes (CNT-SPE). Later, the electrode was modified with EDC/NHS coupling chemistry to produce an amine layer, to adsorb the SARS-CoV-2 spike protein antibody. The sensor exhibited a linear response between 1.0 pg/mL and 10 ng/mL, with an LOD of ∼0.7 pg/mL. A platform for electrochemical sensing was fabricated by Curti et al. [21] employing SWCNT-SPEs, which were functionalized with a DNA aptamer that was already redox-tagged. In the presence of the SARS-CoV-2 spike protein S1, the concentration-dependent folding of a DNA aptamer occurred, which resulted in a change in the amperometric signal with an LOD of 7 nM. Monoclonal antibodies were employed for the functionalization of SWCNT by Li et al. [22], to develop an extremely sensitive immuno-resistive sensor for the detection of SARS-CoV-2. To minimize the contact resistance, silver electrodes were screen-printed onto SWCNTs, and the complete arrangement was mounted on polyethylene terephthalate (PET) film. The LOD of the developed sensor was 350 genome equivalents/mL. Through systematic analysis, Kim et al. [23] examined the relationships between different thin-film characteristics and the sensitivity of CNT thin-film-based immunosensors for the rapid detection of the SARS-CoV-2 virus. They found that smaller surface roughness and better CNT alignment resulted in improved sensitivity at a given film thickness, with a LOD value of 5.62 fg/mL, because of the enhanced bioreceptor-binding surface area. Table 1 summarizes the earlier discussion of the creation of CNT-based biosensors to identify the SARS-CoV-2 virus.

### 3.2. Dengue Virus (DNV)

**Figure 6:**
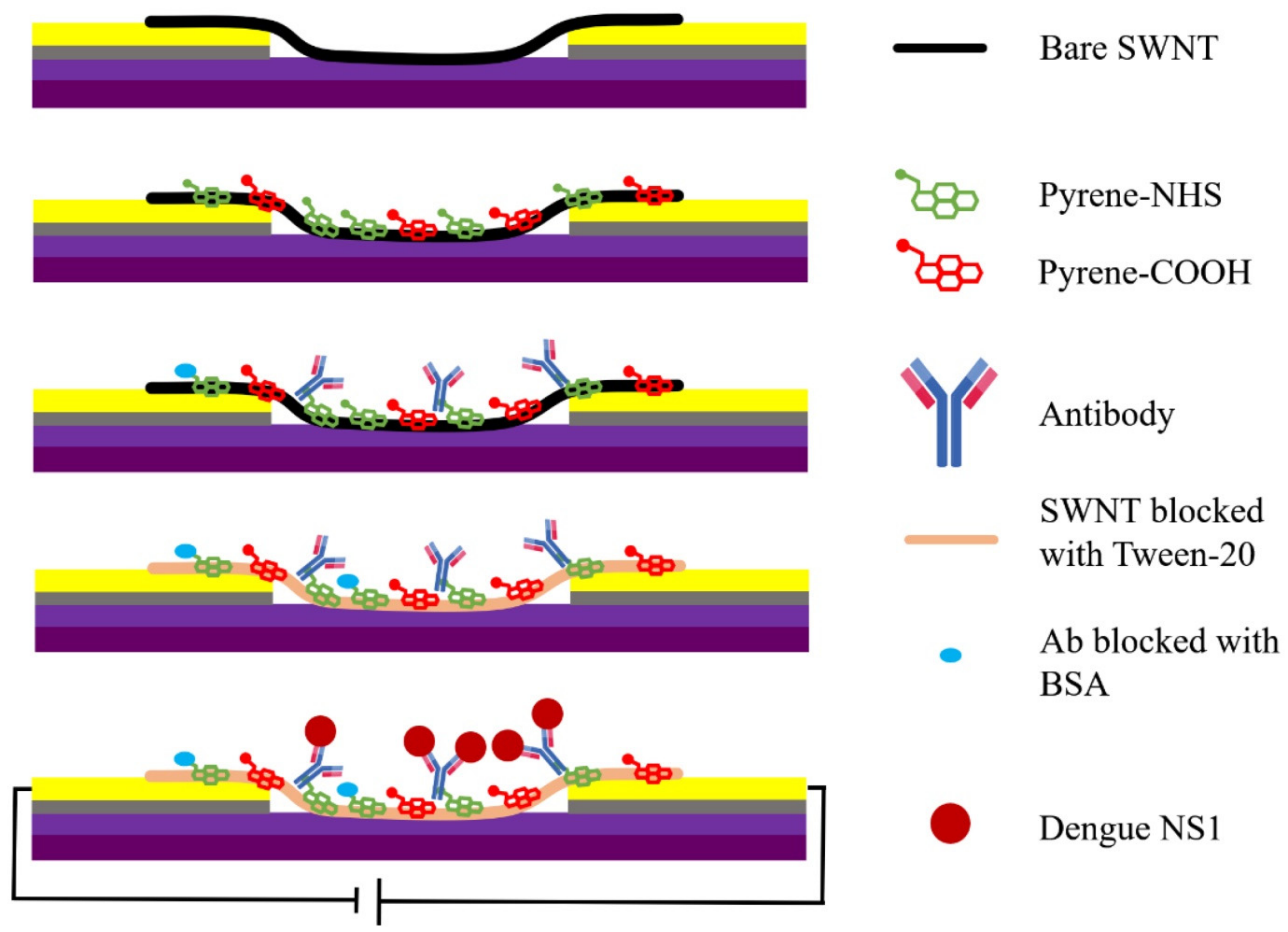
Schematic of the functionalization of SWNT networks with the Dengue Virus (DNV) NS1 antibody (Reprinted with permission from Ref. [28]).

Based on CNT-SPE, an immunosensor for non-structural protein 1 (NS1) of the DNV was created by Dias et al. [24]. They employed a uniform mixture of carboxylated CNTs distributed in carbon ink to make the CNT-SPE and used an ethylenediamine film to covalently attach anti-NS1 antibodies to CNT-SPE. The developed biosensor was able to detect the DNV NS1 protein, with an LOD of 12 ng/mL. A robust poly (allylamine) (PAH) sandwich-based immunosensor was fabricated by Silva et al. [25] for detecting DNV NS1. A thin coating of PAH on carboxylated CNTs helped to immobilize anti-NS1 antibodies on the electrode surface. To strongly bond CNTs to the electrode surface, as well as anti-NS1 antibodies, through their Fc terminus to prevent random immobilization, PAH, a cationic polymer, was used. The fabricated immunosensor had a linear range of operation between 0.1 g/mL and 2.5 g/mL, with an LOD of 0.035 g/mL. An SWCNT-based, inexpensive, label-free chemiresistive biosensor was designed by Wasik et al. [26], where, for the first time, heparin was utilized as a biorecognition component as opposed to a conventional antibody. The biosensor revealed clinically significant sensitivity for people infected by *Aedes aegypti*, concerning the detection of whole DNV, with an LOD of 8 DNV/chip. Later, they modified the biosensor by employing a network of anti-dengue NS1 monoclonal antibodies for the functionalization of SWCNTs, instead of using herpin [27]. The modified biosensor exhibited a linear response in the range of 0.03–1200 ng/mL, with an LOD of 0.09 ng/mL. Almost all laboratory and commercial dengue NS1 diagnostic measures include a blood-drawing procedure, which limits the advantage of point-of-care (POC) diagnostics and reduces patient readiness. Instead of blood, NS1 can be extracted from human saliva for the early detection of dengue infection, which is a straightforward, non-invasive, pain-free, and economical process that can be performed by even untrained/less-trained workers. This pathway was also explored by Wasik et al. [28] through the fabrication of a label-free chemiresistive immunosensor employing a network of anti-dengue NS1 monoclonal antibody-functionalized SWCNTs. The biosensor exhibited a detection range of ~1 ng/mL to 1000 ng/mL for the DNV NS1 protein (Figure 6). To detect DNV antibodies, a high-performance impedimetric immunosensor was developed by Palomar et al. [29] via the deposition of CNT on electrodes and was later functionalized with polypyrrole-NHS to immobilize the DNV 2 NS1 glycoprotein via covalent amide coupling. This biosensor exhibited high linearity after optimization, in a broad range of concentrations (10^−13^ to 10^−5^ g/mL). For the detection of dengue toxin, the CNT/Au nanoparticle (AuNP) composite was deposited on a homemade Au electrode by Palomar et al. [30], to immobilize dengue antibodies on AuNPs through covalent bonding. The electrochemical signal enhancement and improvement in overall performance were achieved due to the porosity of the tri-dimensional network of Au-CNT, with an LOD of 3 × 10^−13^ g/mL. Mendonça et al. [31] fabricated a label-free immunosensor employing a thin film of CNT-ethylenediamine. The covalent immobilization of the anti-NS1 monoclonal antibodies on CNTs allowed for great measurement stability. Finally, differential pulse voltammetry (DPV) was used to analyze the responses to dengue NS1. The measurement showed that the linear range for the operation of the biosensor was 20 to 800 ng/mL, with an LOD value of 6.8 ng/mL. The preceding discussion on the development of CNT-based biosensors for DNV detection is summarized in Table 1.

### 3.3. Influenza Virus

**Figure 7:**
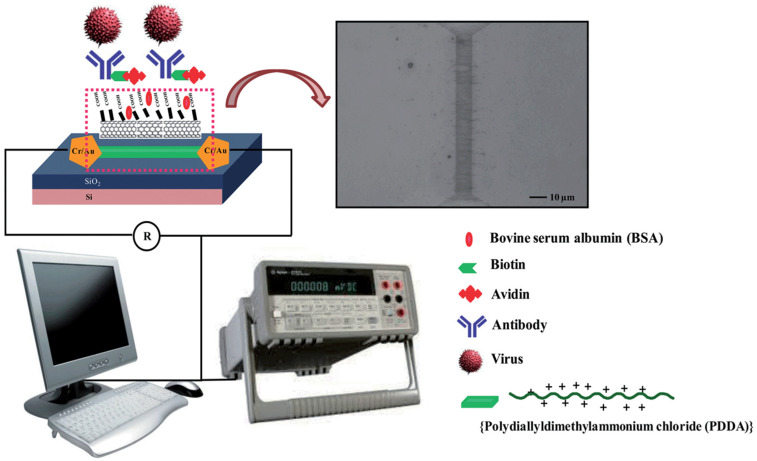
Schematic illustration of the SWCNT-based immunosensor system for H1N1 virus detection. The inset shows an optical image of di-electrophoretically deposited SWCNTs on a PDDA self-assembled monolayer (Reprinted with permission from Ref. [36]).

An MWCNT-cobalt phthalocyanine nanocomposite and poly (amidoamine) (PAMAM) dendrimer were deposited on a glassy carbon electrode (GCE) by Zhu et al. [32] to electrochemically detect the avian influenza virus (AIV) genotype in a label-free format. The DNA probes were, then, effectively immobilized on the modified electrode using the coupling agent, G4 PAMAM dendrimer, and were monitored with DPV to achieve an LOD of 1.0 pg/mL. The surface of MWCNTs was utilized by Tam et al. [33] to immobilize the DNA probe by covalent interactions between the DNA sequence’s amine and phosphate groups. Changes in conductance of the sensor surface were used to detect the hybridization of the DNA probe and the influenza viral DNA for the label-free detection of the influenza virus with an LOD of 0.5 nM. CNT-SPEs were used by Bonanni et al. [34] as an electrochemical-sensing platform for the detection of influenza viral DNA. The CNT-SPEs were functionalized with carboxylic molecules to covalently immobilize the oligonucleotide probe, employing facile carbodiimide chemistry. A direct coupling and sandwich scheme, two distinct techniques for the impedimetric detection of DNA hybridization, were employed and compared. The sandwich scheme revealed better results, with an LOD value of 7.5 fM. A self-assembled SWCNT thin film was prepared by Lee et al. [35] for the fabrication of an affordable, label-free biosensor to detect the swine influenza virus (SIV). The anti-SIV antibodies were bounded with the SWCNT thin film for the detection of SIV, with an LOD of 180 TCID_50_/mL, by monitoring the change in resistance (up to 12%). Dielectrophoretic and electrostatic forces were used to deposit COOH-functionalized SWCNTs on a self-assembled monolayer of the polyelectrolyte, polydiallyl dimethyl-ammonium chloride (PDDA), by Singh et al. [36]. Viral antibodies were immobilized, utilizing biotin-avidin coupling, after avidin was coated on the PDDA-SWCNT channels (Figure 7). Changes in the channels’ resistance were monitored to detect influenza viruses, with a detection limit of 1 PFU/mL. AuNP-decorated CNTs (Au-CNTs) were produced, utilizing phytochemical composites at room temperature, and were employed by Lee et al. to build a plasmon-assisted fluoro-immunoassay (PAFI) for the detection of the influenza virus [37]. The surfaces of Au-CNTs and CdTe quantum dots (CdTe-QDs), the photoluminescence intensity of which varied according to viral concentration, were conjugated with specific antibodies against the influenza virus to achieve an LOD of 1 ng/mL (for Beijing/262/95 (H1N1)), 0.1 pg/mL (for New Caledonia/20/99IvR116 (H1N1)), and 50 PFU/mL (for Yokohama/110/2009 (H3N2)). Later, a two-step method was adopted by Lee et al. [38] to decorate CNTs with Au/magnetic nanoparticles, which offered superior magnetic properties and high electrical conductivity. Later, the Au nanoparticle’s surface was conjugated with thiol-group-functionalized probe DNA to detect influenza viral DNA with a detection limit of approximately 8.4 pM. AuNPs were bonded to the CNT surface via in situ accumulation under mild conditions by Ahmed et al. [39]. The improved peroxidase-like activity of the Au-CNT nanohybrid was employed to develop a supersensitive colorimetric optical sensor to detect the influenza virus. The sensor comprised a test system containing specific influenza antibodies, Au-CNT nanohybrids, 3, 3′, 5, 5′-tetramethyl-benzidine (TMB), and H_2_O_2_. The color of the test system turned blue upon the addition of the influenza virus and the LOD was 3.4 PFU/mL. Fu et al. [40] fabricated chemiresistor-type biosensors to detect AIV, employing semiconducting SWCNTs or nitrogen-doped MWCNTs and non-covalently functionalizing them with DNA probe sequences. Complementary DNA target sequences of AIV, with concentrations ranging from 2 pM to 2 nM, could be detected by the constructed biosensor after 15 min at room temperature. To detect influenza type A viral DNA, a CNT-FETs-based DNA sensor was developed by Tran et al. [41]. The initial probe DNA, the hybridization period, and the reaction temperature were some of the aspects that were examined since they affected the sensing data. The DNA sensor demonstrated a quick response time of under a minute, with a very low (1 pM) detection limit and a broad linear detection range of 1 pM to 10 nM. Wang et al. [42] compared the effectiveness of the aptamer and antibody, concerning the detection of influenza A virus (California/07/2009 (pdmH1N1)), employing an MWCNT-Au conjugated sensing surface with a di-electrode. They found that the electric response and affinity of aptamers were much stronger than those of antibodies. Wang et al. also reported that the aptamer could provide an LOD in the range of 10 fM, whereas the antibody showed an LOD of 1 pM in detecting the influenza A virus. Using flexible and stable SP-CNT-polydimethylsiloxane electrodes, a paper-based immunosensor for the label-free hemagglutinin antigen (HA) detection of several AIVs (H5N1, H7N9, and H9N2) was demonstrated by Lee et al. [43], whereby immune responses were measured via DPV. The LOD values for the different viruses were 55.7 pg/mL for H5N1 HA, 99.6 pg/mL for H7N9 HA, and 54.0 pg/mL for H9N2 HA. In Table 1, an overview of the CNT-based biosensors for influenza detection is provided.

### 3.4. Human Immunodeficiency Virus (HIV)

**Figure 8:**
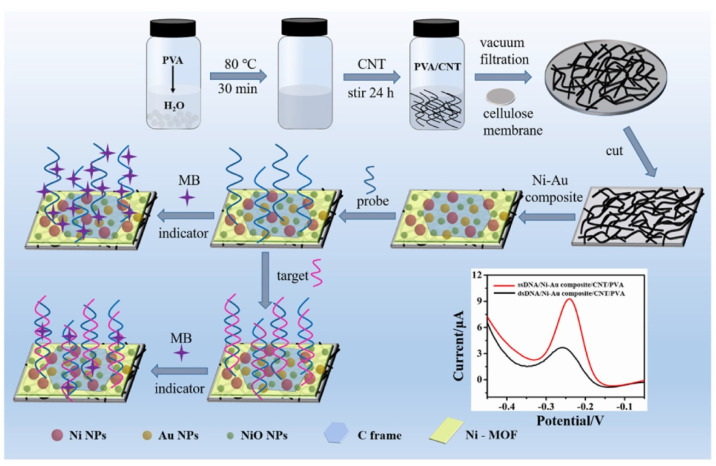
Schematic of the fabrication process for a flexible Ni-Au composite/CNT/PVA film electrode and the detection of the target Human Immunodeficiency Virus (HIV) DNA (Reprinted with permission from Ref. [50].

Mahmoud et al. [44] immobilized thiol-terminated ferrocene-pepstatin (ThFcP) conjugate on an SWCNT/AuNP-modified Au electrode. Electrochemical impedance spectroscopy (EIS) was used to track the nature of the interaction between HIV-1 protease and the ThFcP conjugate, which resulted in the change of the interfacial characteristics of Au electrodes. The electrochemical biosensor was able to detect HIV-1 protease, even at a 10 pM level. Later, thiolated SWCNT/AuNPs were used to modify the disposable SP-Au electrode surface, while ThFcP was subsequently self-assembled on those surfaces by Mahmoud et al. [45] to fabricate a sensitive electrochemical biosensor to detect HIV-1 protease. A nanocomposite of AuNP, amino-functionalized MWCNT, and acetone-extracted propolis (AEP) was prepared by Kheiri et al. [46] and deposited in the same way on an Au electrode for immobilization of the p24 antibody (anti-p24 Ab) to create an immunosensor for the detection of the HIV antigen. The immunosensor demonstrated high electrochemical sensitivity in detecting p24 in a range of concentration from 0.01 to 60.00 ng/mL, with an LOD of 0.0064 ng/mL. An advanced molecularly imprinted polymers (MIPs) electrochemical sensor was fabricated on MWCNT-modified GCE by Ma et al. [47] through the polymerization of the surface, using acrylamide (AAM), N,N′-methylene bisacrylamide (MBA), and ammonium persulphate (APS) as the functional monomer, cross-linking agent, and initiator, respectively. The developed sensor was capable of detecting HIV-p24 in human serum samples, with an LOD of 0.083 pg/cm^3^. A chitosan/glutaraldehyde crosslinking system was employed by Giannetto et al. [48] to immobilize the target protein on disposable CNT-SPE for the maximum exposure of p24, to interact with a mouse anti-p24 IgG1. The linear operating range of the immunosensor was 10 pM to 1 nM, with an LOD of 2 pM, for HIV-related p24 capsid protein in human serum. Harvey et al. [49] observed that denatured proteins can improve the optical responsiveness of CNTs to nucleic acids. Their study revealed that following hybridization, hydrophobic regions of the denatured protein interact with the surface of the CNTs, which results in a larger shift in the nanotube emission. They later employed this strategy for the detection of intact HIV in serum. A nickel-organic composite/AuNP/CNT/polyvinyl alcohol (PVA) substance was used to fabricate a flexible paper-based electrode by Lu et al. [50] for the detection of HIV DNA (Figure 8). The methylene blue was employed as a redox indicator for DNA hybridization on the electrode. The large surface area and the presence of π-electron, donated by the Ni-Au composite, facilitate the higher loading of target DNA. With a linear range of 10 nM–1 μM and an LOD of 0.13 nM, this flexible paper electrode demonstrated good sensing capability. The previous discussion on the creation of CNT-based biosensors to identify HIV is summarized in Table 1.

### 3.5. Hepatitis Virus

**Figure 9:**
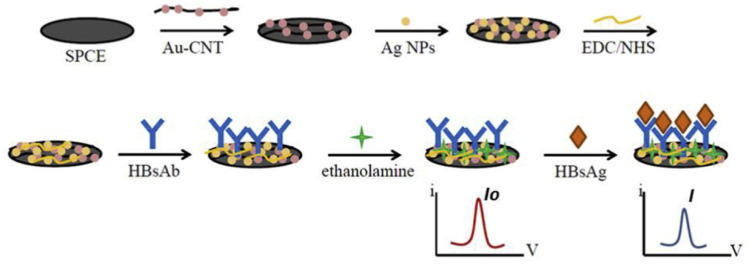
The stepwise fabrication process of the immunosensor for the detection of Hepatitis B virus (HBV) (Reprinted with permission from Ref. [58].

To detect the short DNA sequences associated with the Hepatitis B virus (HBV), a label-free electrochemical DNA biosensor was developed by Li et al. [51], employing 4,4′-diaminoazobenzene (4,4′-DAAB) and MWCNT-modified GCE. The CNT carboxyl groups were covalently linked to the oligonucleotides and the DPV was used to monitor the hybridization reaction, with an LOD of 1.1 × 10^−8^ M. To detect HBV, Oh et al. [52] created an FET-based biosensor, using CNTs consisting of a microfluidic channel with immobilized hepatitis B antibody on it. The electrical conductance changed over time, owing to the presence of the hepatitis B antigen in the channel. The change in the channel conductance was proportional to the hepatitis B antigen concentration. Ly et al. [53] immobilized bovine IgG, employing cyclic voltammetry on a DNA-linked CNT electrode to fabricate an electrochemical biosensor for the detection of human HBV in non-treated blood. The relative standard deviation of 0.2 mL HBV was 0.04 (*n* = 4) within the working limits of 0.035–0.242 mg/mL anti-bovine IgG. A CNT-conducting polymer (CP) network was created by Hu et al. [54] via drop-casting a CNT solution on a GCE, followed by the electrochemical polymerization of a poly (pyrrole propionic acid) (pPPA) film for crosslinking and stabilizing the CNTs. The CNTs served as the network’s structural foundation and provided excellent specific surface areas for immobilizing antibodies. Moreover, owing to its self-limiting growth characteristic, the conducting film facilitated CNT in forming a stable network and offered ample carboxyl groups to immobilize the probe proteins for the detection of hepatitis B surface antigen in serum, with an LOD of 0.01 ng/mL. Amino-CNT and hyaluronic acid (HA) were bonded with amide groups and assembled onto the surface of GCE by Cabral et al. [55]; the response of the electrode in the presence of hepatitis B core protein antibodies was measured by square-wave voltammetry (SWV). The immunosensor response was linear up to 6.0 ng/mL, with an LOD of 0.03 ng/mL. For the detection of the core hepatitis B antigen, an electrochemical immunosensor based on polytyramine (PTy)-CNT composite was developed by Trindade et al. [56]. Because of the substantial creation of NH3^+^ ionic species, the composite possesses high catalytic activity. The HBV was electrochemically identified by SWV in a label-free and reagent-free manner. The immunosensor exhibited a LOD of 0.89 ng/mL while operating in a linear range of 1.0 to 5.0 ng/mL. An AuNPs/chitosan-ferrocene-ammoniated MWCNT (CS-Fc-AMWNT) nanocomposite was prepared by Chen et al. [57] via the Schiff base reaction to achieve a large specific surface area, adequate conductivity, and exceptional biocompatibility. The electrochemical deposition was used to modify the AuNPs for the screen-printed electrode (SPE), while physical adsorption was used to adhere the CS-Fc-AMWNTs composite to the electrode surface. Later, through glutaraldehyde cross-linking, hepatitis B surface antibodies were immobilized on the surface of the electrode. The biosensor can be operated in the range of 1–250 ng/mL for the detection of hepatitis B antigen, with an LOD of 0.26 ng/mL. The SP carbon electrode was modified by Upan et al. [58], through the addition of CNTs that were embellished with AuNP and AgNP (Figure 9). The AuNPs offered biocompatibility and a wide surface area for the immobilization of the hepatitis B surface antibody, which aided the signal improvement. Subsequently, in DPV detection, AgNPs served as a sensing probe to detect the target antigen in the linear range of 1–40 ng/mL with an LOD of 0.86 ng/mL. Using a peptide nucleic acid-functionalized SWCNT-FET biosensor, Dastagir et al. [59] also exhibited the explicit and label-free detection of a hepatitis C virus RNA sequence, with a detection limit of 0.5 pM. The deposition precipitation approach was used by Pusomjit et al. [60] to create Pt nanoparticles that were later coated onto SWCNTs. The generated nanocomposite was finally used as a substrate to immobilize antibodies on a paper-based, SP graphene electrode surface for the purposes of analyzing the hepatitis C virus. DPV was used to measure the target antigen in the range of 0.05 to 1000 pg/mL, with an LOD value of 0.015 pg/mL. Table 1 summarises the preceding discussion on the development of CNT-based biosensors and their use to detect several types of human viruses HBV.

**Table 1:** CNT-based biosensors for human virus detection.

Virus.		Target	Sensor Type	Limit of Detection (LOD)	Detection Range	Detection Platform	Ref. No
SARS-CoV-2		Antigen	FET-based	4.12 fg/mL	0.1 fg/mL to 5.0 pg/mL	CNT surface	[18]
		Antigen	FET-based	0.55 fg/mL	5.5 fg/mL to 5.5 pg/mL	CNT surface	[17]
		Gene	FET-based	10 fM	NA	CNT surface	[19]
		Protein	Optical	12.6 nM	NA	SWCNT substrate	[16]
		Antibody	Electrochemical	0.7 pg/mL	0.7 pg/mL and 10.0 ng/mL	Electrode	[20]
		Protein	Electrochemical	7 nM	NA	Electrode	[21]
		Protein	Immunosensor	350 genome equivalents/mL	NA	Electrode	[22]
		Protein	Immunosensor	5.62 fg/mL	NA	CNT thin-film	[23]
Dengue		Antibody	Immunosensor	10^−13^ g/mL	10^−13^ to 10^−5^ g/mL	Electrode	[29]
		Protein	Immunosensor	12 ng/mL	40 ng/mL to 2 µg/mL	Electrode	[24]
		Protein	Immunosensor	6.8 ng/mL	20 to 800 ng/mL	CNT surface	[31]
		Protein	Immunosensor	0.035 μg/mL	0.1 to 2.5 μg/mL	Electrode	[25]
		Virus	Immunosensor	8 DNV /chip	NA	Electrode	[26]
		Antibody	Immunosensor	0.09 ng/mL	0.03 to 1200 ng/mL	Electrode	[27]
		Protein	Immunosensor	1 ng/mL	1 to 1000 ng/mL	Electrode	[28]
		Toxin	Electrochemical	3 × 10^−13^ g/mL	0.001 to 2 μg/mL	Electrode	[30]
Influenza		Virus	Immunosensor	1 ng/mL (for Beijing/262/95 (H1N1)), 0.1 pg/mL (for New Caledonia/20/ 99IvR116 (H1N1)) and 50 PFU/mL (for Yokohama/110/2009 (H3N2))	50 to 10,000 PFU/mLm (for Yokohama/110/2009 (H3N2))	Au-CNT surface	[37]
		Aptamer and antibody	Immunosensor	10 fM	NA	Au-CNT surface	[42]
		Antibody	Immunosensor	180 TCID_50_/mL	NA	CNT surface	[35]
		DNA	Immunosensor	2 pM	2 to 200 pM	Semiconducting SWCNTs or nitrogen-doped MWCNTs	[40]
		DNA	FET-based	1 pM	1 pM to 10 nM	CNT surface	[41]
		DNA	Immunosensor	8.4 pM	1 pM to 10 nM	Electrode	[38]
		DNA	Electrochemical	0.5 nM	NA	Electrode	[33]
		Virus	Immunosensor	1 PFU/mL	1 to 10,000 PFU/mL	CNT surface	[36]
		Virus	Optical	3.4 PFU/mL	3.4 to 10 PFU/mL	Au-CNT surface	[39]
		DNA	Electrochemical	7.5 fM	NA	Electrode	[34]
		Antigen	Immunosensor	55.7 pg/mL for H5N1 HA, 99.6 pg/mL for H7N9 HA, and 54.0 pg/mL for H9N2 HA	100 pg/mL to 100 ng/m	Electrode	[43]
		DNA	Electrochemical	1.0 pg/mL	0.01 to 500 ng/mL	Electrode	[32]
HIV		Virus	Electrochemical	0.083 pg/ cm^3^	1.0 × 10^−4^ to 2 ng/cm^3^	Electrode	[47]
		Antigen	Immunosensor	0.0064 ng/mL	0.01 to 60.00 ng/mL	Electrode	[46]
		Protease Enzyme	Electrochemical	NA	NA	Electrode	[45]
		DNA	Electrochemical	0.13 nM	10 nM to 1 μM	Electrode	[50]
		Protease Enzyme	Electrochemical	NA	10 pM	Electrode	[44]
		Viral nucleic acids	Optical	NA	NA	Fluorescence Detector	[49]
		Protein	Immunosensor	2 pM	10 pM to 1 nM	Electrode	[48]
Hepatitis	B	Antibody	Immunosensor	0.89 ng/mL	1.0 to 5.0 ng/mL	Electrode	[56]
		Antigen	FET-based	NA	NA	CNT surface	[52]
		Virus	Electrochemical	NA	NA	Electrode	[53]
		Antigen	Immunosensor	0.26 ng/mL	1–250 ng/mL	Electrode	[57]
		Antibody	Immunosensor	0.03 ng/mL	0.03 to 6.0 ng/mL	Electrode	[55]
		Antigen	Immunosensor	0.01 ng/mL	NA	Electrode	[54]
		DNA	Electrochemical	1.1 × 10^−8^ M	7.94 × 10^−8^ to 1.58 × 10^−6^ M	Electrode	[51]
		Antigen	Immunosensor	0.86 ng/mL	1–40 ng/mL	Electrode	[58]
	C	Antigen	Immunosensor	0.015 pg/mL	0.05 to 1000 pg/mL	Electrode	[60]
		RNA	FET-based	0.5 pM	NA	CNT surface	[59]

## 4. Current Challenges and Future Perspectives

Carbon nanomaterials are frequently employed in biomedical applications, due to their multifunctionality and minimal complexity in surface modification, which, in turn, improves their biophysical characteristics. CNT is the sp^2^ hybridized allotrope of carbon, with a hollow cylindrical tubular structure, and has a high aspect ratio. The efficiency and precision of detection in sensing viral genomes, proteins, and other viral cellular biological components using CNT are governed by the exotic characteristics of the nanotubes. CNTs differ from conventional nanomaterials in that they possess many novel physiochemical properties that hold great promise for a variety of applications, including biosensing. It is envisaged that the utilization of CNT’s special capabilities in a biological setting would lead to significant improvements in disease diagnosis, monitoring, and treatment. Consequently, CNT-based biosensors offer many benefits over other types of sensors, such as those based on metal oxides or silicon, including high sensitivity, quick response times, reduced redox reaction potential, and longer lifetimes with greater stability. Although CNTs have many desirable qualities and benefits, dispersion, which is triggered by the high surface energy of the CNTs, stands in the way of moving forward. Because of their extreme hydrophobicity, CNTs cannot be dissolved in water or other common solvents. In order to increase their solubility and other functional qualities, CNTs must be functionalized, depending on the application. The benefits and drawbacks of employing CNTs in biological applications are listed in Table 2.

To establish a faithful nano-bio interface, CNTs may be engineered by means of covalent or non-covalent modification, EDC/NHS chemistry, click chemistry, and length-location tuning. Numerous viruses can go into a dormant stage called latency, wherein they remain inactive inside the host cell before activation [62,63,64,65,66]. Often, it is necessary to identify the virus, even in the latent stage, to eliminate the probability of infection/reinfection. Thus, the pathways may be searched through the functionalization or surface modification of CNTs, to enable CNTs to detect the virus in both the dominant and latent stages. Although the great sensitivity and extended durability of CNTs-based viral biosensors make them a promising candidate for viral detection, their accessibility and the economic perspective are essential for rapid diagnosis. The supervision of economic feasibility is an important component of commercialization. The manufacturing complexity levels should be kept to a minimum from the beginning of the design process, by means of the selection of facile fabrication methods that can be easily scaled up. The selection of materials is also very important, particularly in terms of balancing the cost and the desired material features for the required application. The CNT exhibits tremendous economic potential, especially in light of recent developments that demonstrate a substantial decrease in the cost of making high-quality CNT, which fosters a silver lining in CNT-based nanodevice fabrication. Wearable biosensors with wireless communication facilities should be introduced to address this problem [67], enabling the patient to benefit from swift analysis and report the findings, to ensure a timely diagnosis. The integration of biosensors with electronic gadgets possessing smart read-out capabilities is turning into a top requirement in contemporary state-of-the-art living, in line with the current technological evolution [68]. Conversely, the toxicity of CNT and the greenness of biosensors are currently the major challenges regarding biocompatibility and sustainability. The toxicity of CNT can be reduced with the use of several techniques, such as tuning the surface defects [69], utilizing native small-molecule drugs [70], and attenuating the CNT length [71]. The greenness of the biosensor may be achieved through the green synthesis of CNT; the entire sensing operation must be performed in a green manner, i.e., using green solvents, green waste management, green power management, etc. [72]. In many cases, it was observed that the viruses mutated very rapidly (e.g., SARS-CoV-2 [73]); thus, it is vital to create dependable and effective methods based on integrated multiple biosensor technology for the quick detection of several mutations of a virus at once [74]. Moreover, CNT can also be used as an antiviral agent to inhibit viruses. By adding protoporphyrin IX to acid-functionalized MWCNTs, Banerjee et al. [75] created porphyrin-conjugated MWCNTs, which significantly reduced the capacity of the Influenza A virus to infect mammalian cells when exposed to visible light. Iannazzo et al. [76] investigated the anti-HIV efficacy of several functionalized MWCNTs. The findings demonstrated that the antiviral activity of functionalized MWCNTs was regulated by their hydrophilic functionality and water dispersibility. Thus, CNT can also be employed in a bimodal “detection-inhibition” role to fight against viruses. Finally, further research into the nanotechnology used in virus detection is needed to achieve novel platforms that might completely revolutionize the current viral identification systems used in clinics.

## Figures and Tables

**Table 2:** Pros and cons of using CNTs for biomedical applications. (Reprinted with permission from Ref. [61].

Pros	Cons
Unique mechanical properties offer in vivo stability Extremely large aspect ratio offers a template for the development of multimodal devices Capacity to readily cross biological barriers; novel delivery systems Unique electrical and semiconducting properties; constitute advanced components for in vivo devices Hollow, fibrous, light structure with different flow dynamics properties; advantageous in vivo transport kinetics Mass production-low cost; attractive for drug development	Nonbiodegradable Large available surface area for protein opsonization As-produced material insoluble in most solvents; needs to be surface-treated preferably by covalent functionalization chemistries to confer aqueous solubility (i.e., biocompatibility) Bundling; large structures with less than optimum biological behavior Healthy tissue tolerance and accumulation; unknown parameters that require toxicological profiling of the material A great variety of CNT types makes standardization and toxicological evaluation cumbersome

## Data Availability

Not applicable.
